# The Comparison of Clinical Epidemiology of Hospitalized Patients with COVID-19 during the Third and Fourth Waves of the Pandemic in Gorgan

**DOI:** 10.1155/2022/9634241

**Published:** 2022-12-31

**Authors:** Samira Eshghinia, Rahmat Allah Sharifi Far, Naghimeh Hajimoradloo, Ali Sinesepehr, Ahmad Sohrsbi, Mousa Imeri, Amir Reza Khodanazar Kalti, Erfan Rezaie Shirazi, Roghieh Golsha

**Affiliations:** ^1^Biochemistry and Metabolic Disorders Research Center, Golestan University of Medical Sciences, Gorgan, Iran; ^2^Infectious Diseases Research Center, Golestan University of Medical Science, Gorgan, Iran; ^3^School of Medicine, Golestan University of Medical Sciences, Gorgan, Iran

## Abstract

**Background:**

COVID-19 has turned into a global public health crisis. This study intended to compare demographic characteristics, disease severity, treatment methods, and clinical outcomes in hospitalized patients with COVID-19 during the third and fourth waves of the pandemic in Golestan Province, Iran.

**Methods:**

In this cross-sectional study, the clinical epidemiology of all COVID-19 patients, who were hospitalized in two educational hospitals in Golestan Province for 30 days from the start of the third and fourth waves of the coronavirus pandemic in 2021-2022, was assessed. Their electronic medical records were used to collect their epidemiological, demographic, laboratory, and clinical information and treatment outcome.

**Results:**

In all, 930 electronic medical records of the hospitalized patients (351 in the third wave and 579 in the fourth wave) were studied. In the third and fourth waves, 29.06% and 13.13% of the patients had severe COVID-19, respectively (*P* = 0.001). The number of deaths in the third wave was larger compared to the fourth wave (*P* = 0.015). The mean duration of hospitalization was longer in the third wave than in the fourth wave (*P* = 0.001). The drugs administered most in these two waves were remdesivir, dexamethasone, and heparin, and the patients who received these drugs were compared in the third and fourth waves (*P* = 0.001).

**Conclusion:**

The reduced rate of mortality in the fourth wave was compared to the third wave. This reduction can be attributed to the change in the national strategy adopted in terms of hospitalization criteria and treatment protocols taking into account the acquired experience, earlier hospitalization, and start of drug therapy.

## 1. Background

Coronavirus disease (COVID-19), which is caused by severe acute respiratory syndrome coronavirus 2 (SARS-CoV-2), has become a global public health crisis [1, 2]. In addition, given the rapid spread of this virus in various countries, the World Health Organization (WHO) declared on January 30, 2020, that COVID-19 was a Public Health Emergency of International Concern (PHEIC) [3]. Up to January 30, 2020, more than 370 million cases of COVID-19 were confirmed, and more than 5.6 million deaths due to this disease were reported. Moreover, the largest number of new cases were reported from the US, France, India, Brazil, and Germany. At present, the current global epidemiology of SARS-CoV-2 is defined by the rapid global spread of the Omicron variant, while all other variants of concern including VOCs (i.e., Alpha, Beta, Gamma, and Delta) and VOIs (i.e., Lambda and Mu) are exhibiting a declining trend in all six WHO regions [4].

In Iran, the first registered case of COVID-19 was detected on February 19, 2020, in Qom City, and other cases were then reported from the other provinces within a short time [5]. There have been 6.5 confirmed cases of COVID-19 and more than 130,000 deaths caused by it in Iran so far. A recent study in Iran showed that about half of the people with a positive polymerase chain reaction (PCR) test for COVID-19 required hospitalization, and almost 5%, mainly with the underlying diseases of diabetes and hypertension, had to be rehospitalized. More than 50% of the hospitalized patients had not been screened by the health system, and the screening had no effect on the length of stay (LOS) and outcomes of the disease [6]. In addition, Baigi et al. reported that older age, being male, history of comorbidities (especially cancer), and decreased consciousness at arrival were among the factors that could considerably increase the risk of death in hospitalized COVID-19 patients in Iran [7].

Increase in the ratio of the number of positive tests to the total number of tested people, hospitalization over the last three days, and the number of critically ill patients are among the important indicators of monitoring the COVID-19 situation and determining the wave of the disease [8]. Based on this definition, the third wave of COVID-19, caused by the usual variant of the virus, happened in Golestan Province in fall 2021, and the fourth wave, with the UK variant as its dominant pathogenic agent, occurred in spring 2022 [9]. The epidemiological characteristics of patients of the first and second waves of the pandemic in Iran have been reported in previous studies [10, 11] and this comparison is not reported for the third and fourth waves. Consequently, this research was conducted to compare demographic characteristics, disease severity, treatment methods, and clinical outcomes in hospitalized COVID-19 patients during the third and fourth waves of the pandemic in Golestan Province, Iran.

## 2. Methods

This cross-sectional study was carried out on 930 COVID-19 patients with positive PCR test results during the third (from November 1, 2020, to December 1, 2020) and fourth (from April 4, 2021, to May 5, 2021) waves of the disease who were all patients over the age of 14 and were hospitalized at the 5-Azar and Sayyad Shirazi Educational Hospitals in Golestan Province. The epidemiological, demographic, laboratory, and clinical information of the patients and their treatment outcomes were extracted from their electronic medical records. The exclusion criteria included being symptomatic patients with negative PCR tests, being patients who were discharged from the hospitals after 24 hours, those having incomplete electronic medical records, and those for whom necessary variables are not recorded.

Laboratory-confirmed COVID-19 cases refer to patients with positive PCR results in nasal and pharyngeal swabs. Based on the latest national coronavirus instructions [12], arterial blood oxygen saturation between 90 and 94% and lung involvement of <50% was considered the moderate case of the disease. Moreover, ≥30 breaths per minute, arterial blood oxygen saturation of <90%, and the need for using NIV (noninvasive ventilation) and HFNO (high-flow nasal oxygen) were classified as severe cases of the disease.

Descriptive analyses of the variables were reported in terms of median (interquartile range (IQR)), number (%), or simple ranges when appropriate. No imputation was made for missing data. When the data were normally distributed, means of the continuous variables were compared using the independent group *t*-test results; however, for the not normally distributed data, the Mann–Whitney test was used. Proportions for the categorical variables were compared using the chi-square test; however, when the data were limited, the Fisher exact test was used. All the statistical analyses were performed using STATA 12.0 software (Stata Corporation, College Station, TX, USA).

This article is the result of a research project approved by the Research Ethics Commission of Golestan University of Medical Sciences (ir.goums.rec.1400.195). Information was recorded using the codes and numbers of electronic medical records, and patients' information was kept confidential.

## 3. Results

The fourth and third waves of the epidemic accounted for 62.3% and 37.7% of the 930 studied patients, respectively (i.e., 579 and 321 patients, respectively). As shown in Table 1, the patients were in the age range of 16–102 years old and the median age of 57 years old (interquartile range, IQR = 26), and 53.9% of them were female. There were no significant differences between the patients in the third and fourth waves in terms of age and gender. The rates of the need for hospitalization in the ICUs and the number of deaths in the third wave were greater than those in the fourth wave. The most common underlying diseases were diabetes and hypertension with 22.5% and 24.7%, respectively, in both waves. The average LOS was longer in the third wave compared to the fourth wave (*P* = 0.001).

As presented in Table 2, the most commonly used antiviral drug in both waves was remdesivir (for 53.84% and 77.54% of the patients in the third and fourth waves, respectively) (*P* = 0.01). The second most common drug was favipiravir, administered to 23.64% and 77.37% of the patients in the third and fourth waves (*P* = 0.02), respectively. Regarding the antibiotics, ceftriaxone (with 36.47%), meropenem (with 33.05%), and ceftazidime (with 24.79%) were the most commonly used antibiotics in the third wave, and ceftriaxone (with 33.51%), ceftazidime (with 32.82%), and cefepime (with 25.73%) were the antibiotics the patients received the most in the fourth wave. The quantities of the other antibiotics used in the third and fourth waves were not statistically significant.

**Figure 1:**
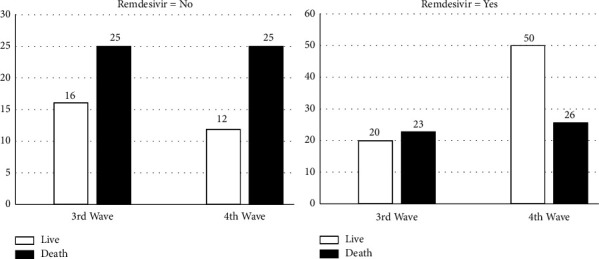
Outcome of remdesivir treatments among third and fourth wave COVID-19 cases during hospitalization.

Figure 1 shows that, in all, 638 of the patients (68.60%) received remdesivir during the both waves. In the fourth wave, this drug was administered more in comparison to the third wave (*P* = 0.001). In the third wave, mortality was lower in patients receiving remdesivir compared to those who did not receive medications; however, this difference was not statistically significant (*P* = 0.336). In the fourth wave, however, mortality was significantly lower (*P* = 0.001) in patients receiving remdesivir compared to those who did not receive medications (6.30% compared to 20.77%).

## 4. Discussion

In this cross-sectional research, the electronic medical records of 930 patients were studied for one month, from the beginning of the third wave with the usual variant to a month after the start of the fourth wave with the dominant UK variant of the coronavirus, at two educational hospitals in Golestan Province located in the northeast part of Iran. The results indicated that the total number of patients hospitalized during the fourth wave was larger than that in the third wave; however, the number of deaths in the fourth wave was significantly smaller compared to that in the third wave. In the fourth wave, given the experience gained from the beginning of the disease to that date, we witnessed changes in the criteria for hospitalization and disease treatment that led to earlier hospitalization and the start of drug therapy with corticosteroids, remdesivir, and heparin. In a similar vein, in other studies conducted in Iran and other countries, the mortality rates were higher in the early waves compared to the later ones. The reasons for this can be attributed to acquiring sufficient understanding of the disease and its treatment during the first wave followed by changes in hospitalization criteria, more rapid admission of critically ill patients, changes in the treatment strategy, and the acquired experience [10, 13]. In contrast, in another study on 2,044,482 patients hospitalized in the first and second waves in South Africa, both the hospitalization cases and the number of deaths significantly increased in the second wave compared to the first wave. The reasons for this increase were probably virus mutation and fatigue among the healthcare workers.

This research revealed that the average LOS of COVID-19 patients in the third wave was longer than that of the patients in the fourth wave. In addition, the need for hospitalization in the ICUs was greater, and the mortality rates were higher in the third wave compared to the fourth one. These results can be attributed to early hospitalization and early start of drug therapy, which reduced the number of patients with severe diseases. Similarly, in a study that compared the patients in the first and second waves in Babol City, the number of severe cases of the disease was significantly larger in the first wave than in the second one, and it was suggested that this happened probably because the mean age of the patients in the first wave was higher, and hence, the number of severe cases of the disease was larger compared to the second one [7, 10]. However, in this research, although the mean age of the patients in the third wave was somewhat higher than the fourth one, this difference was not statistically significant and could be due to changes in hospitalization criteria and the treatment received by the patients in the fourth wave (i.e., the patients were hospitalized earlier and received drug treatment, and hence, the disease progression towards severity was slower in the fourth wave). As for LOS, the average LOS in this research was significantly longer in the third wave compared to the fourth one, probably because severe cases were more common in the third wave, and patients with lower SpO2 were hospitalized in the third wave compared to the fourth wave.

In both third and fourth waves, older age and comorbidities such as hypertension, diabetes, and cardiovascular disease in COVID-19 patients were significantly more common reasons of death. Likewise, in the research conducted by Mithal et al., severe cases of the disease and mortality resulting from COVID-19 in diabetes patients were significantly more common compared to nondiabetic patients [14]. Diabetes was reported as a risk factor for disease severity and severe outcomes in another study, whereas hypertension was not related to the severity of complications and mortality [15]. In a cohort study on more than 300,000 COVID-19 patients, comorbidities, including pulmonary diseases and hypertension, were among the main causes of increased mortality, especially in the elderly [16].

In this research, the most common comorbidities in both waves were hypertension, diabetes, and cardiovascular diseases in that order, and they were more common in the third wave than in the fourth one. The American Heart Association has stated that viral diseases including COVID-19 can increase the risk of heart attacks in people who have plaques in their blood vessels [17]. Research results have shown that viral diseases can make it more likely for a piece of the plaque coating the vessels to break off and block blood flow to the heart [18]. Diabetes and high blood sugar levels increase the ability of the virus to grow in the human body. In addition, diabetes increases inflammation and weakens the immune system, which in turn decreases the ability to cope with infectious diseases [19]. In agreement with the results of this research, a review study in China, which studied 4659 patients concerning disease outcomes, reported that hypertension, diabetes, and cardiovascular diseases were the most common underlying diseases in COVID-19 patients [20].

In this study, remdesivir was used more in the fourth wave which was due to the change in the start indication of the drug. Early administration of this drug may also be one of the reasons for the reduced mortality rate in the fourth wave. In agreement with these results, a double-blind trial of remdesivir in 1062 hospitalized COVID-19 patients showed that, compared to the placebo, remdesivir reduced the recovery period in patients and was less frequently accompanied by serious complications such as respiratory system infections [21]. However, in a meta-analysis performed on recent studies in this area, the effect of remdesivir on reducing mortality rate and other outcomes of the disease was not confirmed [22]. In conformity with the results of our research, the results of a recent review study also indicated the positive effects that early administration of antiviral drugs before severe inflammation had [23]. In this research, favipiravir was used more in the third wave, although it was reported in a study that this drug influenced the recovery rate of patients with mild to moderate disease severity [24]. However, the results of other studies showed that administration of this drug did not result in reduction in the rate of hospitalization in ICUs, intubation, or mortality [25]. Honarvar et al. also examined 2835 acute respiratory distress syndrome patients for epidemiological and clinical features. Older age, blood oxygen level, headache, and comorbidities including cardiovascular, respiratory distress, diabetes, chronic lung and kidney disease, and cancer were associated with more risk of death among patients with 2019-nCoV[26].

The cross-sectional nature of the data in this research disrupts the evaluation of causal associations and the determination of causal direction. To overcome these limitations, it is necessary to use prospective data to conduct causal studies on similar hypotheses. In addition, the studied population included only patients hospitalized in educational hospitals in Golestan Province, which limited the generalizability of the results to the population of all hospitalized patients, which is another limitation of this study.

## 5. Conclusion

The findings of this research indicated that the number of hospitalized patients was larger in the fourth wave, while the number of deaths was smaller in the fourth wave compared to the third one. This could be due to the change in hospitalization criteria and treatment protocols that led to early hospitalization and drug therapy. Moreover, being older than 50 and having an underlying medical condition such as hypertension and diabetes are associated with increased mortality rates. Considering the results of this research, we suggest hospitalization of the patients in the early stages of the disease and when PsO2 reaches 94% and drug therapy (the drugs are remdesivir, dexamethasone, and heparin) begins as early as possible.

## Figures and Tables

**Table 1:** Epidemiological and clinical characteristics among third and fourth wave COVID-19 cases.

Variables	Total (*n* = 930)	Third wave (*n* = 351)	Fourth wave (*n* = 579)	*P* value
Hospital duration, mean (SD), day	5.61 (5.40)	6.50 (6.32)	5.06 (4.67)	**0.001**
Age, mean (SD), years old	55.37 (17.13)	56.39 (17.64)	54.75 (16.80)	0.156
Age range (years old), *n* (%)				
<20	9 (1%)	3 (0.9%)	6 (1%)	0.585
20–35	115 (12.4%)	44 (12.5%)	71 (12.3%)	
36–50	252 (27.1%)	86 (24.5%)	166 (28.7%)	
51–65	288 (31%)	106 (30.2%)	182 (31.4%)	
66–80	193 (20.8%)	81 (23.1%)	112 (19.3%)	
>80	73 (7.8%)	31 (8.8%)	42 (7.3%)	
Gender, *n* (%)				
Female	501(53.9%)	180 (51.3%)	321 (55.4%)	0.223
Male	429 (46.1%)	171 (48.7%)	258 (44.6%)	
Admitted to ICU, *n* (%)				
Yes	178 (19.1%)	102 (29.1%)	76 (13.1%)	**0.001**
No	752 (80.9%)	249 (70.9%)	503 (86.9%)	
Outcome, *n* (%)				
Recovered	821 (88.3%)	299 (85.2%)	520 (90.4%)	**0.01**
Death	109 (11.7%)	52 (14.8%)	55 (9.6%)	
Diabetes, *n* (%)	209 (22.5%)	91 (25.9%)	118 (20.3%)	**0.03**
Cardiovascular disease, *n* (%)	70 (7.5%)	30 (8.5%)	40 (6.9%)	0.21
Chronic lung disease, *n* (%)	38 (4.0%)	23 (6.5%)	15 (2.5%)	0.56
Hypertension, *n* (%)	230 (24.7%)	101 (28.7%)	129 (22.2%)	**0.01**
Chronic kidney disease, *n* (%)	26 (2.7%)	19 (5.4%)	7 (1.2%)	**0.001**
Cancer, *n* (%)	19 (2.0%)	8 (2.2%)	11 (1.9%)	0.43
COPD, *n* (%)	24 (2.5%)	17 (4.8%)	7 (1.2%)	**0.008**
CNS, *n* (%)	25 (2.6%)	16 (4.5%)	9 (1.5%)	**0.006**

Bold values mean *p* < 0.05 (Statistical significance).

**Table 2:** Comparison of medication prescribing among third and fourth wave COVID-19 cases.

Variables	Total (*n* = 930)	Third wave (*n* = 351)	Fourth wave (*n* = 579)	*P* value
Remdesivir	638 (68.60%)	189 (53.84%)	449 (77.54%)	**0.001**
Favipiravir	531 (57.09%)	83 (23.64%)	448 (77.37%)	**0.02**
Dexamethasone	773 (83.11%)	260 (74.07%)	513 (88.60%)	**0.001**
Methylprednisolone	172 (18.49%)	77 (21.93%)	95 (16.49%)	**0.03**
Heparin	695 (74.73%)	220 (62.67%)	475 (82.03%)	**0.001**
Ceftriaxone	322 (34.62%)	128 (36.46%)	194 (33.50%)	**0.04**
Meropenem	222 (23.87%)	116 (33.04%)	106 (18.30%)	**0.001**
Ceftazidime	277 (29.78%)	87 (24.78%)	190 (32.81%)	**0.009**
Cefepime	210 (22.58%)	61 (17.37%)	149 (25.73%)	**0.03**
Levofloxacin	117 (25.58%)	24 (6.83%)	93 (16.06%)	**0.001**

Bold values mean *p* < 0.05 (Statistical significance).

## Data Availability

The COVID-19 Golestan data used to support the findings of this study are available from the corresponding author upon request.
